# Heat stress affects tassel development and reduces the kernel number of summer maize

**DOI:** 10.3389/fpls.2023.1186921

**Published:** 2023-06-07

**Authors:** Pan Liu, Baozhong Yin, Limin Gu, Shaoyun Zhang, Jianhong Ren, Yandong Wang, Weiwei Duan, Wenchao Zhen

**Affiliations:** ^1^ College of Agronomy, Hebei Agricultural University, Baoding, China; ^2^ Key Laboratory of North China Water saving Agriculture, Ministry of Agriculture and Rural Affairs, Baoding, China; ^3^ College of Plant Protection, Hebei Agricultural University, Baoding, China; ^4^ State Key Laboratory of North China Crop Improvement and Regulation, Baoding, China

**Keywords:** heat stress, maize, tassel, kernel number per ear, anther

## Abstract

Maize grain yield is drastically reduced by heat stress (HTS) during anthesis and early grain filling. However, the mechanism of HTS in reproductive organs and kernel numbers remains poorly understood. From 2018 to 2020, two maize varieties (ND372, heat tolerant; and XY335, heat sensitive) and two temperature regimens (HTS, heat stress; and CK, natural control) were evaluated, resulting in four treatments (372CK, 372HTS, 335CK, and 335HTS). HTS was applied from the nine-leaf stage (V9) to the anthesis stage. Various morphological traits and physiological activities of the tassels, anthers, and pollen from the two varieties were evaluated to determine their correlation with kernel count. The results showed that HTS reduced the number of florets, tassel volume, and tassel length, but increased the number of tassel branches. HTS accelerates tassel degradation and reduces pollen weight, quantity, and viability. Deformation and reduction in length and volume due to HTS were observed in both the Nongda 372 (ND372) and Xianyu 335 (XY335) varieties, with the average reductions being 22.9% and 35.2%, respectively. The morphology of the anthers changed more conspicuously in XY335 maize. The number of kernels per spike was reduced in the HTS group compared with the CK group, with the ND372 and XY335 varieties showing reductions of 47.3% and 59.3%, respectively. The main factors underlying the decrease in yield caused by HTS were reductions in pollen quantity and weight, tassel rachis, and branch length. HTS had a greater effect on the anther shape, pollen viability, and phenotype of XY335 than on those of ND372. HTS had a greater impact on anther morphology, pollen viability, and the phenotype of XY335 but had no influence on the appearance or dissemination of pollen from tassel.

## 1 Introduction

Since 1880, the average global temperature has increased by around 0.8°C, with two-thirds of this increase occurring since 1975, at a rate of approximately 0.2°C per decade. By 2100, the global average surface temperature is expected to rise by 2–3°C (Apostolatos et al., 2010; Tian et al., 2018). The frequent occurrence of extreme heat in mid- and low-latitude regions has caused severe damage to crops, seriously affecting global food security (Tesfaye et al., 2016; Zandalinas et al., 2017; Libecap and Dinar, 2022). Pollination is an important aspect of the growth and development of higher plants and is significantly affected by unfavorable environments (Ali et al., 2018). Heat stress (HTS) is a typical environmental adversity that has a significant impact on both the physiological functions and phenotypic characteristics of plant reproductive organs and the pollination process (Hatfield and Prueger, 2015; Gabaldón-Leal et al., 2016). Brief HTS during the critical flowering stage can cause significant yield loss (Tian et al., 2018; Wang et al., 2022).

Maize *(Zea mays* L.), an annual plant belonging to the family Gramineae, is a highly valuable food and forage crop and is sensitive to heat (Yin et al., 2021; Wang et al., 2022). Maize yield is affected by HTS during all growth stages, with plants being most vulnerable during the flowering stage (Rattalino Edreira et al., 2011; Feng and Hao, 2020). The morphology and physiological functions of tassels, the reproductive organs for pollen production, are sensitive to HTS (Schoper et al., 1987). Pollen shedding is dramatically reduced at > 36°C owing to failed anther dehiscence, and pollen viability is greatly reduced at > 38°C because of the disturbed pollen structure and components in maize (Wang et al., 2019). Plants exposed to HTS from the nine-leaf stage (V9) to the tasseling stage (VT) show stunted tassel growth, disrupted anther structure, reduced pollen viability, and a shortened pollination period (Shao et al., 2021).

During the anthesis stage, HTS had a less pronounced effect on kernel weight than on kernel quantity (Suwa et al., 2010; Wang et al., 2019). Wang et al. (2019) found that the kernel weight of plants (including during the silking stage) exposed to high temperatures for 14 days [i.e., 40°C (day) and 30°C (night)] was comparable to that of plants exposed to slightly lower temperatures [i.e., 32°C (day) and 22°C (night. However, HTS significantly reduced the kernel per spike. Liu et al. (2022) reported that, under HTS, both before and after flowering, the number of kernels per spike decreased by 73%–98% on average. Similar results were obtained when the temperature was increased from the V9 to the VT. Studies have been conducted on the effects of HTS on kernel number and yield, as well as on the morphology and physiological functions of reproductive organs. However, the process by which HTS affects reproductive organ development and kernel formation is complex (Cairns et al., 2013; Lizaso et al., 2018) and is influenced not only by the timing of HTS coinciding with maize fertility, but also by factors such as the intensity and duration of HTS and varietal characteristics (Shao et al., 2021).

China is one of the world’s leading maize-producing regions, accounting for over one-fifth of global maize production. The North China Plain (NCP) is China’s primary maize-producing region and is responsible for approximately 40% of the country’s total output (Li et al., 2022). The tasseling–anthesis stage of summer maize in NCP coincides with frequent HTS periods in the region (late July to mid-August), resulting in bad seed-setting and yield reduction (SHAO et al., 2021). Therefore, understanding the disaster mechanism of HTS is imperative to the development of a stress-resistant, high-yield cultivation technology system for maize in the NCP.

Using maize varieties with various heat sensitivities, a simulated HTS experiment was established in a greenhouse during tassel development over a 3-year period in the northern part of the NCP with the aims of (i) clarifying the effects of HTS on the phenotypic characteristics of tassels and anthers, pollen microstructure, and dispersal properties of summer maize, and (ii) determining if HTS reduces the number of kernels per ear, and its correlation with changes in tassel phenotype, pollen properties, and anther structure.

## 2 Materials and methods

### 2.1 Overview of test site

**Figure 1:**
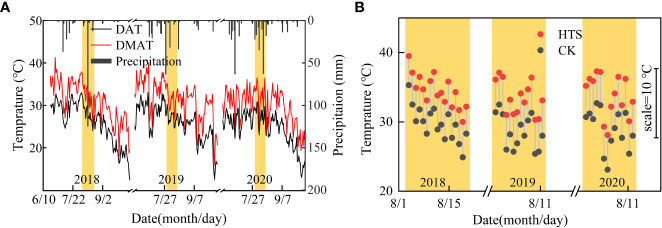
Daily temperature and precipitation during the experiment. **(A)** Daily average temprature (DAT) and daily maximum temprature (DMAT), and precipitation under natural conditions. **(B)** Average temperature between 8:00 and 16:00 in the greenhouse treated with HTS and the average temperature under natural conditions during the same time period.

This study was performed at the Xinji Experimental Station of Hebei Agricultural University, north of the NCP (Mazhuang Village, Xinji City, Hebei Province, China, 115.22° E, 37.92° N, 43 m above sea level) throughout four summer maize-growing seasons (from mid to late June to early October, from 2018 to 2020). From 1981 to 2017, the average annual precipitation and temperature at this station were 470.3 mm and 12.9°C, respectively. The average annual precipitation and temperature during the summer seasons were 345.0 mm and 25.2°C, respectively. Daily precipitation as well as daily maximum, minimum, and average temperatures from 2018 to 2020 are shown in **Figure 1A**. At the experimental station, the topsoil was a neutral loam with a pH of 7.4. The organic matter content of the 0–20 cm soil layer was 18.5 g kg^–1^, and the corresponding total nitrogen, alkali nitrogen, available phosphorus, and potassium contents were 1.0 g kg^–1^, 91.6 mg kg^–1^, 56.9 mg kg^–1^, and 231.2 mg kg^–1^, respectively. The 0–20 cm soil layer contained 18.5 g kg^–1^ organic matter, 1.0 g kg^–1^ total nitrogen, 91.6 mg kg^–1^ alkali nitrogen, 56.9 mg kg^–1^ available phosphorus, and 231.2 mg kg^–1^ potassium.

### 2.2 Experimental design and treatments

**Figure 2:**
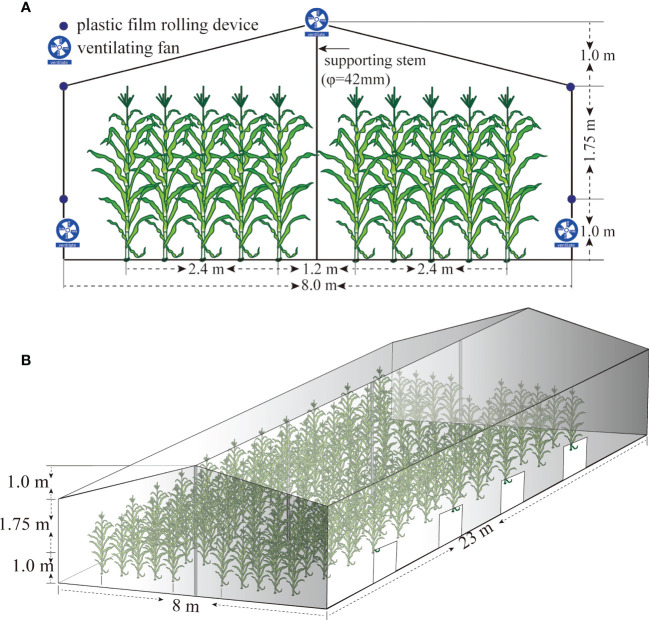
Simulated diagram of temperature increase in greenhouse maize. **(A)** Schematic diagram of the front elevation of the shed. **(B)** Schematic diagram of the 45° direction of the side elevation of the shed.

A two-factor split-zone design was adopted, and the main treatments were HTS and natural control (CK), with side treatments applied to maize varieties Xianyu 335 (XY335, a heat-sensitive variety) and Nongda 372 (ND372, a heat-tolerant variety). The four treatments (372CK, 372HTS, 335CK, and 335HTS) were repeated thrice. The CK group showed normal growth under natural conditions with an area of 66.6 m^2^ per plot. A greenhouse heating system was used in the HTS treatment group, with an area of 18.0 m^2^ (3.0 m width × 6.0 m length). The shed floor was 8.0 m wide, the heights of both sides were 2.75 m, the height of the middle was 3.75 m, and the length was 23.0 m. The top and surrounding areas of the shed were covered with a polyolefin (PO) plastic film (transmittance > 95%). There were 1.0-m-high ventilation belts on both sides of the shed. The airflow volume was varied by adjusting the height of the plastic film covered by the rotating shaft. To regulate the temperature inside the shed, exhaust systems were placed on both sides (**Figures 2A, B** **)**.

The daily HTS treatment time was 8 h (8:00–16:00), and the HTS treatment began at V9 (recorded as DAT0) and continued until the end of the anthesis stage. The specific HTS process was as follows: all the PO plastic films were in place for HTS at 8:00, and the films were removed for cooling at 16:00. The greenhouse temperature was 5 ± 0.5°C higher than that in natural conditions during the HTS treatment period in the test (**Figure 1B**). The plastic film covering the shed was rolled up when the maize was in its natural growth stage outside the HTS phase. If rain occurred during the HTS period, the same volume of supplemental irrigation was applied after the cessation of the rain to maintain uniform soil moisture levels across all treatments. The seeds were sown on 21 June 2018, 16 June 2019, and 2020, with a row spacing of 60 cm and a planting density of 6,000 plants hm^–2^. With the application of 112.5 kg N hm^–2^ at the bottom of seeding, P_2_O_5_ and K_2_O at 150.0 kg hm^–2^ and 75.0 kg hm^–2^, respectively, were applied as basal fertilizer. Furthermore, 112.5 kg N hm^–2^ as urea was applied together with the first irrigation in spring. Irrigation was maintained at 45 mm every year after sowing.

### 2.3 Phenotypic index of tassel

During the 2018–2020 growing season, 10 representative plants in each group were selected pre-V9 and labeled prior to HTS treatment and the extraction of tassels. On days 8 (DAT8), 12 (DAT12), 16 (DAT16), and 20 (DAT20) after HTS, the number of branches (TBN), main axis length (MAL), branch axis length (BAL), main axis and branch florets of the tassel (MFN and BFN, respectively), and tassel volume (TV_s_) of the selected plants were measured. To avoid the impact of observation and growth determination, two groups of maize samples were selected alternately.

**Figure 3:**
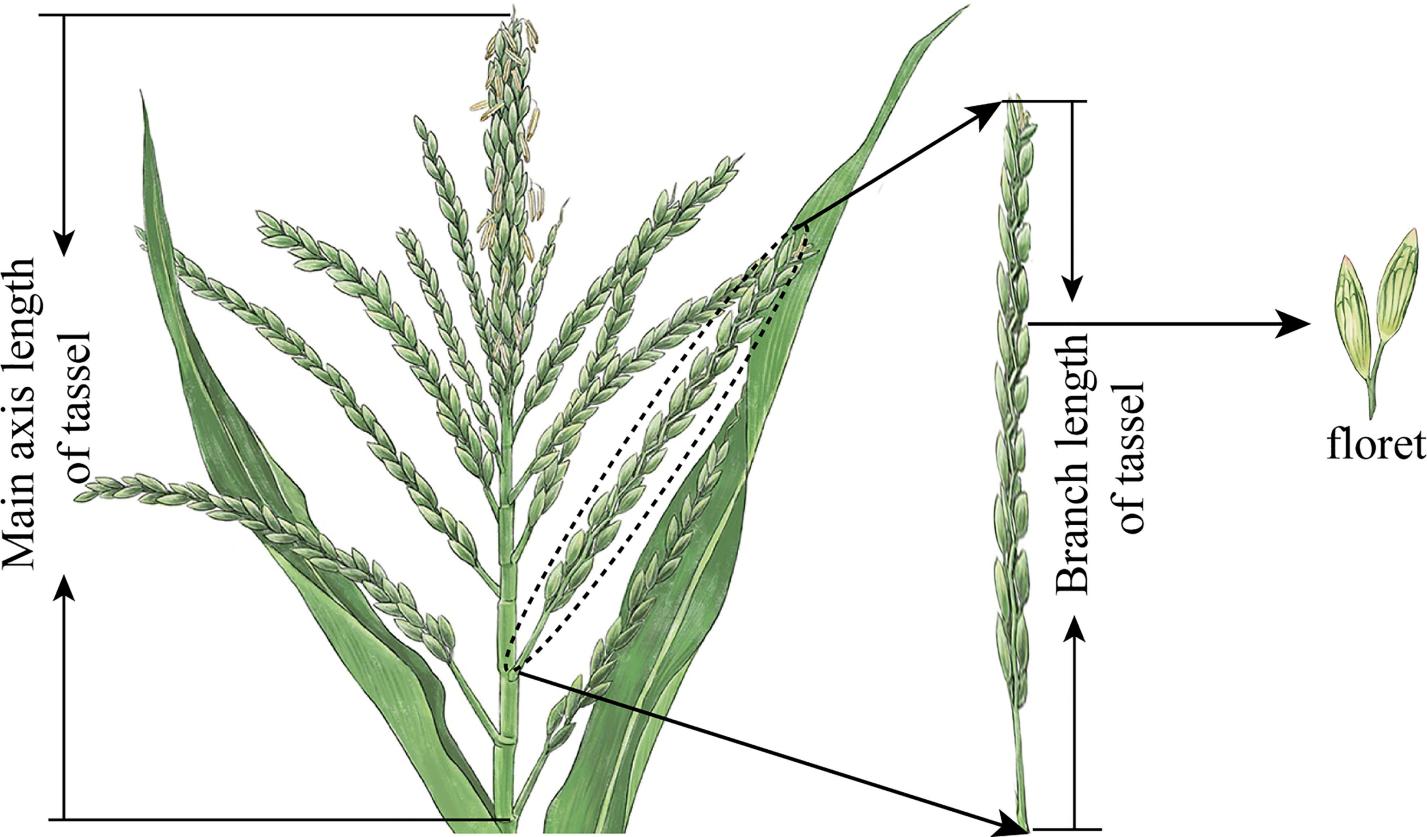
Measurement standard of main axis and branch length of maize tassels and schematic diagram of florets.

The total number of branches in a tassel is the sum of all branches with a branch length of less than 1.5 cm. Plants with dead limbs were excluded. The MAL (cm) of the tassel is the total distance between the lowest point of the main axis and the tip of the spike, and the BAL (cm) is the distance between the lowest point of the branch and the tip of the branch (**Figure 3**). MFN is the total number of florets along the main axis of the tassel, and BFN is the total number of florets along a single branch (count plant^–1^). Three representative plants were selected for each treatment: the part above the base of the first branch of the tassel was cut off, and all branches and main axes were cut into 3- to 5-cm segments. The tassel volume (TV_s_) (cm^3^) per plant was measured using the displacement method.

### 2.4 Pollen dispersal quantity and pollen weight of tassel

During the 2018–2020 growing season, 10 plants were chosen as representatives for the daily monitoring of tassel extraction and floret opening. When the tassels and florets were about to open, the tassel was covered with a polyvinyl chloride (PVC) funnel [10 cm higher than the tassel, and the base was sealed with polytetrafluoroethylene (PTFE) tape] to cover the tassels and collect pollen daily at approximately 16:00 h. The pollen, anther microstructure, and pollen vitality were measured separately from the anthers and debris.

**Figure 4:**
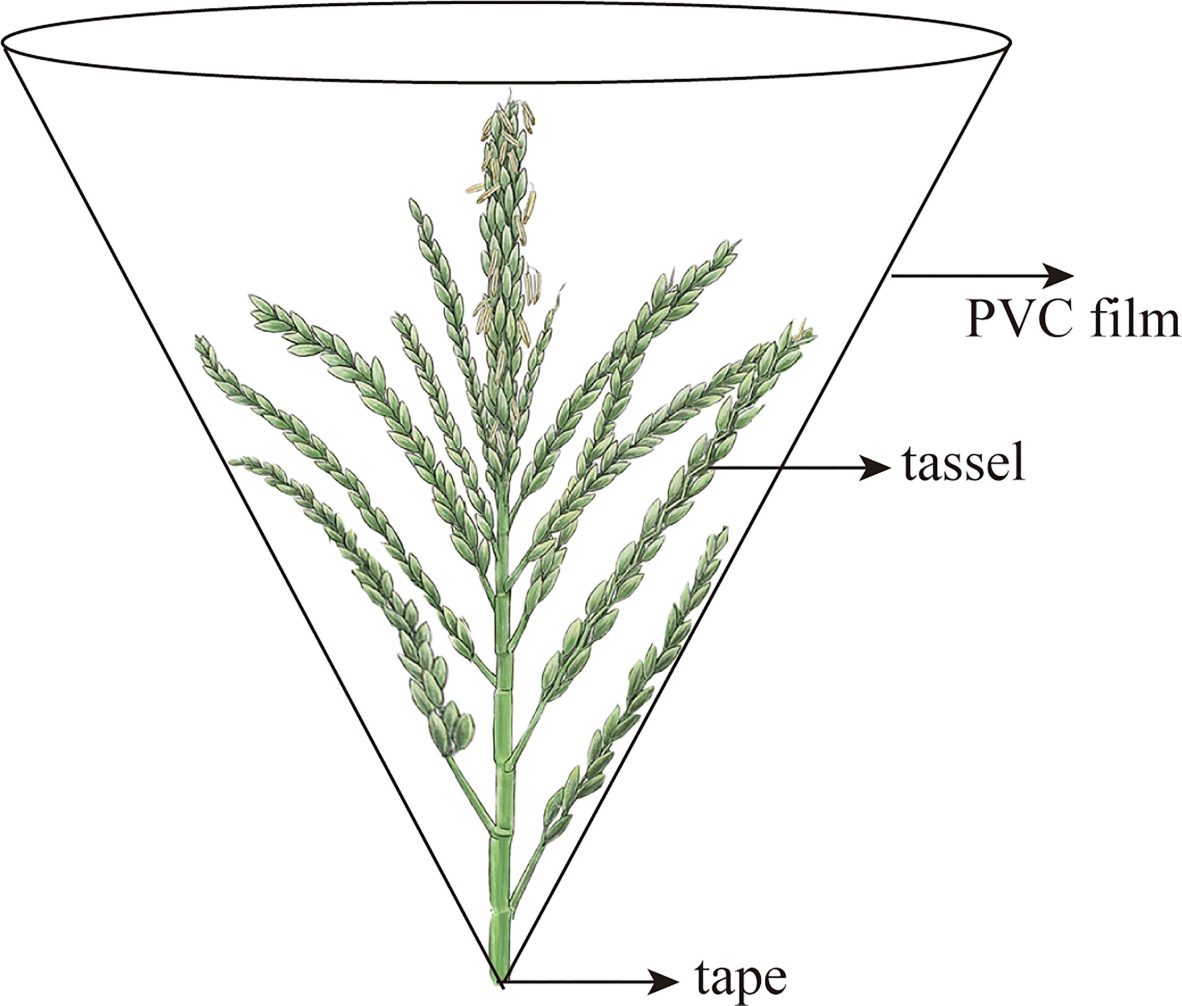
Maize pollen collection device.

Before tassel extraction in the 2018–2020 growing season, 10 plants that underwent the same growth process and exhibited the same growth trends were selected for marking each time, and the progress of tassel extraction and floret opening was observed daily. When it was observed that the tassels and florets were about to open, a PVC funnel was used to cover the tassels (Wang et al., 2019); the top of the film was approximately 10 cm higher than the tassel top, and the base was sealed with PTFE tape (**Figure 4**). To avoid damaging the tassels and stems, two holes were made on each side of the top of the film, through which a rope was passed to fix the film. Pollen was collected daily from the funnel-shaped film at approximately 16:00 h, carefully separated from the anthers and debris, and then weighed.

### 2.5 Pollen, anther microstructure, and pollen vitality

Between 9:00 and 10:00 on days 3 and 4 after anthesis in 2018, five plants with uniform growth were selected from each treatment and their fresh anthers and pollen were collected. The length and volume of the anthers and pollen vitality were measured, and the ultrastructure of the pollen was observed (Begcy and Dresselhaus, 2017).

(1) Anther length (AL_s_) and volume (AV_s_). Anthers were of natural length (cm) from the base to the top. The anther volume was measured using the drainage method (cm^3^).
(2) Anther microstructure. Anthers were placed overnight in Formalin-Aceto-Alcohol fixative soution (FAA) fixed solution for fixation, dehydrated with ethanol gradient, made xylene transparent, embedded in paraffin, and sliced to 8 μm thickness. The sealed film was made permanent and observed and photographed using an Olympus-DP71 optical microscope.
(3) Pollen vitality. The pollens were placed on a clean slide and stained with 0.5% triphenyl tetrazole chloride. After 10–15 min, the pollens were observed under a stereomicroscope to determine their color, and photographed. The pollens that were stained red were marked as active. The proportion of active pollens to the total number of pollens in each field was defined as the percentage pollen vitality (%) (Wang et al., 2021).
(4) Pollen microstructure. Pollens without anthers were collected, fixed with a 3% glutaraldehyde solution, rinsed with phosphoric acid buffer, dehydrated with an acetone gradient, placed on a sample table, vacuumed, sprayed with a gold coating, and observed using a KYKY-2800B scanning electron microscope (Wang et al., 2019).

### 2.6 Kernel number per ear

At the late stage of waxy ripening, sample sections with relatively consistent growth in each treatment were regularly collected from five plants to measure the kernel number of all ears.

### 2.7 Data processing and statistical analysis

Analysis of variance (ANOVA) was performed using SPSS 26.0 (IBM, NY, USA). Data are presented as means ± standard error (SE). One-way ANOVA was conducted, and a Student’s *t*-test was used to compare treatment means at the 5% level. Pearson’s correlation analysis was performed using SPSS software (version 26.0).

## 3 Results

### 3.1 Effect of HTS on effective branchs number and tassel volume

#### 3.1.1 Branchs number of tassel

In addition to DAT8, HTS, variety, and year, the interaction between year and temperature significantly affected the number of branches in the tassel (*p *< 0.05). At DAT8–16, the number of effective spikelet branches of the two varieties in the CK group increased, whereas at DAT16–20, the number either remained unchanged or decreased (decaying branches were not counted). Under HTS, the tassel index showed a gradual increase at DAT8–12 and a gradual decrease at DAT12–20. Compared with the CK group, it was observed that tassel branching in the early stage was promoted in the two varieties under the HTS treatment at DAT8–12, whereas at DAT16, inhibition was observed. Compared with other varieties, the numbers of spikelets in the CK and HTS ND372 treatments were higher than that of the XY335 treatment (74% and 76% higher, respectively, 3-year average).

#### 3.1.2 Tassel volume

Except for DAT12 and DAT16, there were significant differences between the years (**Table 1**). In addition, excluding DAT12, the effect of HTS on the TV_s_ was significant, whereas the influence of year (Y) × treatment (T) was negligible. With an increase in the number of HTS days, the volume of tassels increased rapidly at the DAT8–16 stage, whereas that at the DAT16–20 stage remained stable or slightly decreased. The TV_s_ of the ND372 variety peaked at DAT16, whereas that of the XY335 variety peaked at DAT12. HTS treatment increased the total volume of 337 varieties (DAT8–12 and XY335) but significantly decreased the total volume of 372 varieties (DAT16–20) and XY335 (DAT12–20). Compared with that of the XY335 variety, the TV_s_ of the ND372 variety was significantly greater under all conditions (21.2% higher on average over the 3 years) and 28.4% and 13.5% higher under natural and HTS conditions, respectively. In general, HTS increased the maximum TBNs by 9.8% and 30.0% relative to the CK treatment for the 372HTS and 335HTS varieties, respectively; however, HTS accelerated the decline of tassels. HTS also reduced the TV_s_ of the 372HTS and 335HTS varieties, by 32.0% and 15.1%, respectively, compared with their CK treatments.

**Table 1:** Branch number and volume of tassels under treatments at different temperatures.

Year	Treatment	Days after treatment (d)							
		Branch number of tassel				Volume of tassel (cm^3^ plant^–1^)			
		8	12	16	20	8	12	16	20
2018	372CK	6.3 b	7.7 b	7.7 b	8.0 a	13.3 b	18.0 a	32.3 a	32.0 a
	372HTS	11.0 a	8.7 a	10 a	8.0 a	19.3 a	20.3 a	20.7 b	17.5 b
	335CK	2.7 c	3.7 d	3.7 c	4.0 a	13.0 b	20.7 a	20.7 b	16.7 b
	335HTS	5.3 b	6.0 c	4.3 c	4.3 a	13.3 b	19.3 a	16.3 b	15.8 b
2019	372CK	5.3 b	10.0 a	11.3 a	11.3 a	9.9 c	17.4 b	31.9 a	31.6 a
	372HTS	9.3 a	10.7 a	8.3 b	8.3 b	17.8 a	19 ab	24.0 b	22.5 b
	335CK	3.3 c	7.0 b	4.3 d	4.3 d	15.5 b	19.2 ab	19.0 c	15.4 c
	335HTS	5.7 b	7.0 b	5.7 c	5.7 c	18.5 a	20.4 a	16.7 d	16.0 c
2020	372CK	6.3 bc	11.7 a	9.7 a	9.7 a	9.2 d	15.8 b	33.5 a	31.1 a
	372HTS	10.0 a	13.0 a	10.3 a	10.3 a	14.6 a	22.4 a	21.8 b	19.4 b
	335CK	4.3 c	9.3 b	6.7 b	6.7 b	12.4 b	20.7 a	22.4 b	19.2 b
	335HTS	6.7 b	12.0 a	3.7 c	3.7 c	11.0 c	17.2 b	17.7 c	16.9 c
Year (Y)		NS	**	**	**	**	NS	NS	*
Treatment (T)		**	**	*	**	**	NS	**	**
Y × T		NS	*	**	**	NS	NS	NS	NS

**P < 0.01; *P < 0.05, NS, not statistically significant at *P* < 0.05. Different lowercase letters indicate significant differences between the treatments in the same year (*P* < 0.05).

### 3.2 Effect of HTS on tassel length

#### 3.2.1 Main axis length of tassel

As shown in **Table 2**, short-term HTS treatment (DAT8) can accelerate tassel growth. The TVs of the XY335 and ND372 varieties were 5.8% and 5.5% higher than the CK treatment, respectively. The MAL of the tassels of the XY335 variety were higher than those of the ND372 variety: 13.6% and 13.9% higher under HTS and CK conditions, respectively (3-year average). From DAT8 to DAT12, tassel MAL was continuing to grow under CK conditions (26.8% on average over the 3 years) but modest under HTS conditions (9.4% mean over the 3 years). Except for 335HTS, the MAL continue to increase during DAT12-16. The MAL growth for the 372CK variety was the highest (11.4%), followed by the 372HTS (5.7%) and 335CK varieties (4.5%; 9.4% mean over the 3 years). By the end of the HTS treatment (DAT20), the MAL of the 372CK and 335CK variety were significantly higher than those of the respective HTS treatments (16.8% and 14.4% higher, respectively).

**Table 2:** Main axis and branch length of tassel under treatments at different temperatures.

Year	Treatment	Days after treatment (d)							
		Main axis length of tassel (cm)				Branch length of tassel (cm)			
		8	12	16	20	8	12	16	20
2018	372CK	17.1 b	21.4 a	26.1 a	30.6 a	35.5 b	66.1 a	70.5 a	76.0 a
	372HTS	17.6 b	20.4 a	25.1 a	25.1 c	45.5 a	56.3 b	62.7 a	62.7 b
	335CK	19.6 a	22.4 a	26.2 a	27.9 b	34.2 b	47.6 c	47.8 b	48.7 c
	335HTS	20.1 a	21.6 a	24.9 a	24.9 c	38.1 b	44.8 c	45.4 b	45.4 c
2019	372CK	16.8 c	24.2 a	29.2 a	30.9 a	37.0 bc	64.2 a	67.8 a	69.6 a
	372HTS	17.7 c	20.8 b	24.6 c	24.9 c	42.4 a	53.2 c	58.7 b	58.6 b
	335CK	19.3 b	23.7 a	27.0 b	28.4 b	34.2 c	57.1 b	57.9 b	58.8 b
	335HTS	21.1 a	21.4 b	25.2 c	25.5 c	38.3 b	44.9 d	49.3 c	49.0 c
2020	372CK	16.3 c	19.9 c	27.8 b	31.0 a	37.7 c	64.3 a	69.2 a	69.7 a
	372HTS	17.8 b	17.7 d	25.1 c	29.2 b	42.4 b	53.6 c	56.0 c	58.2 c
	335CK	18.3 b	24.3 a	28.8 a	29.4 b	32.5 d	59.0 b	62.3 b	62.8 b
	335HTS	19.2 a	22.0 b	24.3 d	24.5 c	45.7 a	49.9 d	50.3 d	50.4 d
Year (Y)		*	**	NS	**	NS	**	NS	NS
Treatment (T)		**	**	**	**	**	**	**	**
Y × T		NS	NS	*	NS	**	**	**	**

**P < 0.01; *P < 0.05, NS, not statistically significant at *P* < 0.05. Different lowercase letters indicate significant differences between the treatments in the same year (*P* < 0.05).

A comparison of the differences between the two cultivars further revealed that the MAL of the ND372 variety was 8.0% and 5.8% greater than that of the XY335 variety under CK and HTS conditions, respectively. Except for DAT16, there were significant or extremely significant differences between years and all treatments. However, the interaction between Y and T was significant only at DAT16.

#### 3.2.2 Branch length of tassel

At the early stages of HTS treatment (DAT8), the BAL of the tassels of the 335CK and 372CK varieties showed no significant difference; however, the shorter HTS treatment period (8 d) accelerated the elongation of the tassel branches, and the tassel branches of the ND372 and XY335 varieties were 18.4% and 21.4% longer than those of plants subjected to the corresponding CK treatments (3-year average) (**Table 2**). With the extension of HTS time, the BAL growth rates of the 372CK and 372HTS varieties were significantly higher than that of the XY335 variety. By DAT16, the BAL of each treatments are basically stable. At this time, the BAL of the 372HTS and 335HTS groups decreased by 14.5% and 13.1% (3-year average), respectively, compared with their respective controls. By the end of the treatment, the BAL of the XY335 variety was lower than that of the ND372 variety, i.e., it was 20.5% and 19.1% lower under the CK and HTS treatments, respectively. By comparing the variation from different sources, it was observed that Y had a significant impact on BAL only at DAT12, while T and Y × T had a significant influence at all measuring times. Overall, HTS significantly reduced the MAL of the tassels by 14.4% (ND372) and 16.6% (XY335), respectively; and reduced the BAL of the tassels by 9.5% (ND372) and 9.8% (XY335), respectively.

### 3.3 Effect of HTS on the number of spikelets

#### 3.3.1 Number of florets in main axis of tassel

With increasing HTS treatment time, the MFN in each treatment group first increased and then decreased, reaching a peak at DAT12. The MFN for each treatment followed the order 372CK > 372HTS > 335CK > 335HTS (**Table 3**). Under natural growth conditions, the MFN of the ND372 variety was significantly higher than that of the XY335 variety (3-year average of 33.6%). HTS significantly reduced the number of MFNs, and the numbers of MFNs of the ND372 and XY335 varieties decreased by 13.4% and 32.5%, respectively, compared with those of the CK group. The number of small flowers on the main axis of the XY335 variety was significantly lower than that of the ND372 variety under both natural and high-temperature treatments. Further comparison of the differences between different sources showed that Y and T were the main sources of the difference in the number of small flowers on the main axis, whereas Y × T had a significant effect only at DAT8.

**Table 3:** Main axis and branch florets of tassels under treatments at different temperatures.

Year	Treatment	Days after treatment (d)							
		Number of florets in main axis of tassel				Number of florets in branch of tassel (cm^3^ plant^–1^)			
		8	12	16	20	8	12	16	20
2018	372CK	244.7 a	295.6 a	246.8 a	242.7 a	149.1 b	184.6 b	239.1 a	227.4 a
	372HTS	208.2 b	227.5 b	206.1 b	205.3 b	156.4 a	168.7 c	185.3 c	190.8 c
	335CK	187.5 c	199.0 c	165.4 c	173.6 c	134.1 c	196.4 a	199.9 b	200.9 b
	335HTS	159.4 d	173.2 d	117.1 d	114.6 d	145.7 b	166.6 c	168.8 d	169.3 d
2019	372CK	233.6 a	287.2 a	255.0 a	254.7 a	160.2 b	240.9 a	228.6 a	226.4 a
	372HTS	205.1 b	240.5 b	217.1 b	219.6 b	192.9 a	200.1 d	203.3 c	204.0 b
	335CK	169.3 c	214.9 c	166.0 c	161.2 c	136.5 d	218.5 b	221.7 b	222.2 a
	335HTS	154.4 d	137.5 d	117.4 d	128.0 d	163.0 b	174.2 d	185.1 d	187.9 b
2020	372CK	228.9 a	284.9 a	233.4 a	214.3 a	148.1 c	228.8 a	230.0 a	234.9 a
	372HTS	183.2 c	237.3 b	212.5 b	206.4 ab	192.2 a	176.0 b	182.4 b	183.8 b
	335CK	206.3 b	240.0 b	206.43 b	204.3 b	130.9 d	181.3 b	186.9 b	185.6 b
	335HTS	158.2 d	172.8 c	126.63 c	117.5 c	156.4 b	157.4 c	162.8 c	168.8 c
Year (Y)		**	**	**	NS		**	**	**
Treatment (T)		**	**	**	**		**	**	**
Y × T		**	NS	NS	NS		**	**	**

**P < 0.01; NS = not statistically significant at *P* < 0.05. Different lowercase letters indicate significant differences between the treatments in the same year (*P* < 0.05).

#### 3.3.2 Number of florets in the branch of tassel

As shown in **Table 3**, the short-term high-temperature treatment (8 d) accelerated the differentiation rate of spikelet branches and florets, and the ND372 and XY335 varieties were 18.4% and 15.9% higher, respectively, than under the corresponding CK treatments (3-year average). When the high-temperature treatment time was increased, the BFN growth rates of the 372CK and 372HTS varieties were significantly higher than those of the XY335 variety. By DAT16, the BFN in each treatment group was stable. The BFN scores of the 372HTS and 335HTS varieties were 18.1% and 15.0% lower, respectively, than those of the corresponding controls (3-year averages). By the end of the treatment, the BFN of the tassels of the XY335 variety was less than that of the ND372 variety, specifically the BFN in the 335CK group was 11.5% less than in the 372CK group, and the BFN in the 335HTS group was 9.1% less than in the 372HTS group (3-year average). A comparison of the variation from different sources showed that Y, T, and Y × T had a significant effect on the BFN at all measurement times. In general, HTS significantly reduced the MFNs and BFNs of tassels in both the ND372 (by 13.5% and 18.2%, respectively) and XY335 groups (by 26.2% and 9.6%, respectively).

### 3.4 Effect of HTS on pollen fresh weight, number, and activity

#### 3.4.1 Daily pollen weight

**Figure 5:**
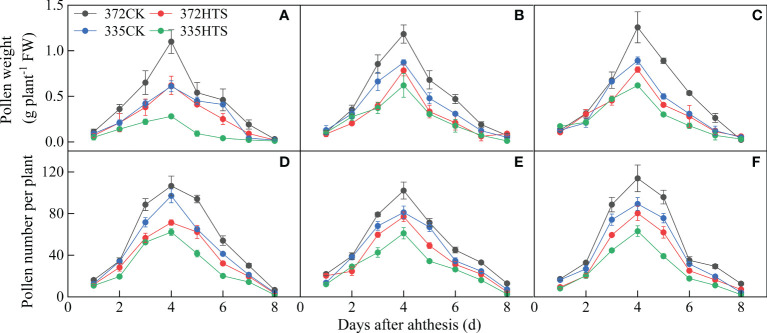
The quantity and weight of maize pollen per plant under different temperatures **(A–C)** depicts the fresh weight of pollen per plant in 2018, 2019, and 2020 respectively. **(D–F)** Number of pollen grains per plant in 2018, 2019, and 2020 respectively.

With an increase in the number of days of pollen dispersal, the fresh weight of pollen (PWs) produced showed a single peak trend, in which it first increased and then decreased, and the PW collected reached its peak on day 4. Under the CK conditions, there was a significant difference in PWs between the two varieties. Among them, the daily PWs of the ND372 and XY335 varieties were 0.04–1.18 g plant^–1^ and 0.04–0.79 g plant^–1^, respectively, and the total PWs were 3.81 g/plant^–1^ and 2.68 g/plant^–1^, respectively. The total and maximum daily PWs of the ND372 variety were 41.9% and 49.0% higher, respectively, than those of the XY335 variety. HTS significantly reduced the maximum PWs and total PWs during athesis period (by 37.0% and 41.0%, respectively). There was no significant difference between the two varieties in the reduction of PW and total PW under HTS conditions, but the daily PW and total PW of the ND372 variety under HTS conditions were significantly higher than those of the XY335 variety (by 45.1% and 38.1%, respectively) (**Figures 5A–C**).

#### 3.4.2 Daily pollen number

The change in the number of maize pollens (PNs) was similar to that of the fresh pollen weight. The daily PNs also showed a single peak trend, first increasing and then decreasing, and reached its peak on day 4. HTS and variety significantly affected the PNs (*p *< 0.01 or *p *< 0.05). The daily average PNs and maximum daily PNs of the two varieties were significantly reduced, by 43.4% and 32.8%, respectively, under the HTS treatment. Under the CK treatment, the daily average PNs and maximum daily PNs of the ND372 variety were 25.9% and 19.95% higher, respectively, than those of the XY335 variety. Under the HTS treatment, the daily average PNs of the two varieties decreased by 24.7%, but the daily average PNs and maximum daily PNs of the ND372 variety were 25.86% and 28.62% higher than those of the XY335 variety, respectively (**Figures 5D–F**). In general, HTS reduced PWs and PNs of maize, but there was no significant difference between the two varieties (the PWs of the ND372 and XY335 varieties decreased by 41.2% and 40.2%, respectively, and the PNs decreased by 32.3% and 35.9%, respectively, based on the 3-year average).

#### 3.4.3 Daily pollen vitality

**Figure 6:**
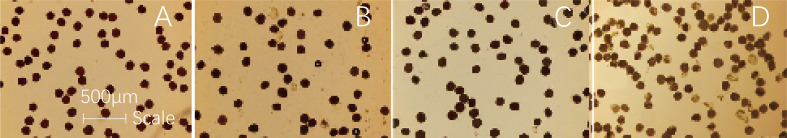
Maize pollen vitality under different temperatures. **(A, B)** are 372CK and 335CK, respectively; **(C, D)** are 372HTS and 335HTS, respectively.

In the present study, the depth of pollen staining represented the level of pollen vitality (PVs). Under CK conditions, the pollen is deeply stained and is of regular size and shape. During observation, the viable pollens of the ND372 variety accounted for 94.3%, and the XY335 variety accounted for 90.0%. The vitality of the XY335 variety was slightly lower than that of the ND372 variety, but the difference was not significant (**Figures 6A, B** **)**. After the HTS treatment, the degree of pollen staining differed significantly between the two varieties. Viable pollens of the ND372 variety accounted for 81.3% of the pollens, whereas that of the XY335 variety accounted for only 71.0% of the pollens. The PVs of the two varieties decreased by 13% and 19%, respectively, compared with those of the CK groups (**Figures 6C, D** **)**. Further comparison of the two varieties subjected to the same HTS treatment revealed that the PVs of the 335HTS variety was 9.7% lower than that of the 372HTS variety. In general, HTS decreased PVs (both varieties decreased by an average of 16%), and the decrease was greater in the heat-sensitive varieties.

### 3.5 Effect of HTS on tassel pollen and anther microstructure

#### 3.5.1 Anther microstructure and volume

**Figure 7:**
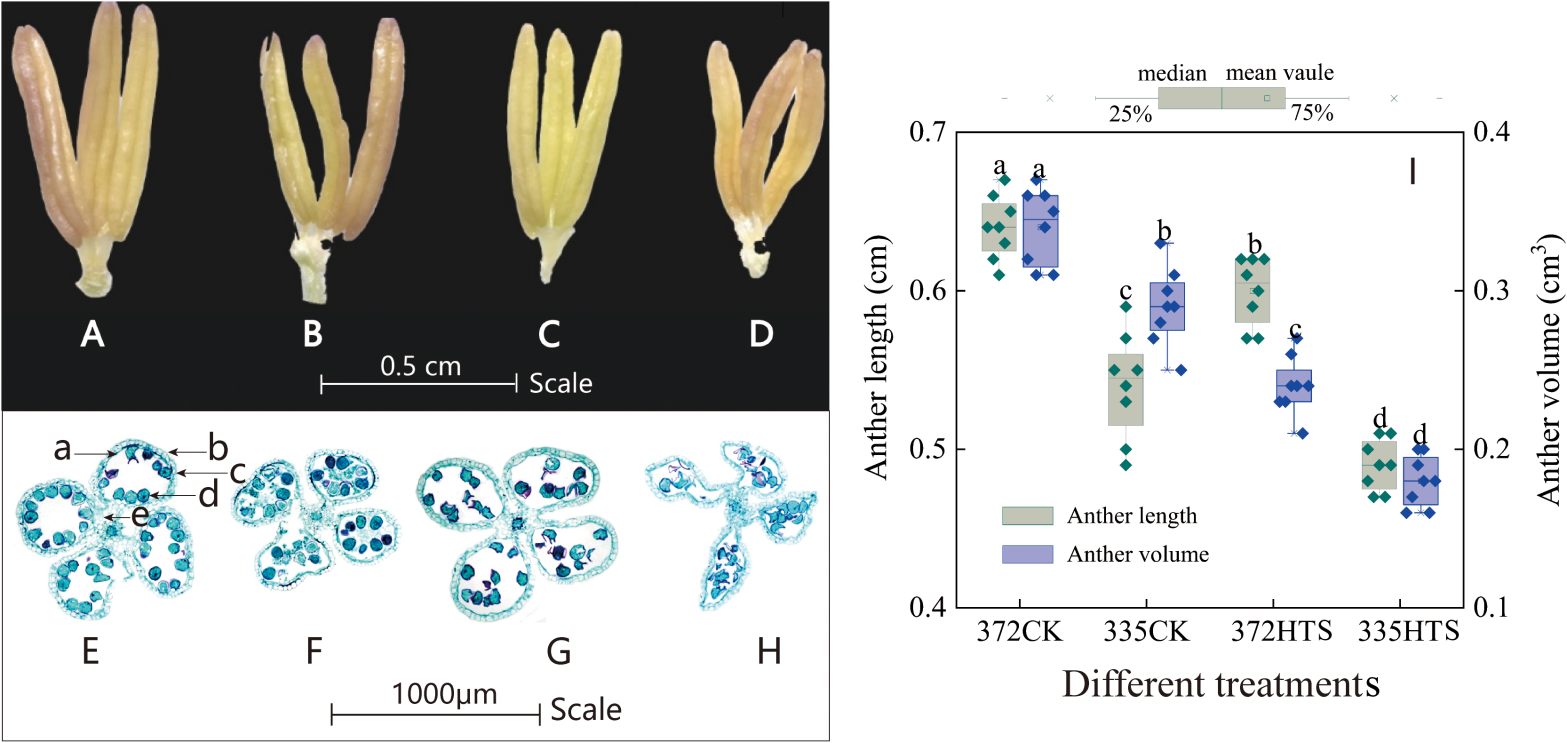
The shape, structure, length, and volume of maize anthers under different temperatures. **(A, E)** are 372CK, **(B, F)** are 335CK, **(C, G)** 372HTS, and **(D, H)** are 335HTS. **(I)** shows the difference in anther length and volume under different temperatures. Different lowercase letters in the data indicate significant differences between treatments of the same index (P<0.05).

At ambient temperature, the anthers of both varieties bulged without any evident deformation. Compared with the other varieties, the ALs and AVs of the ND372 variety under natural conditions were 6.3% and 14.7% higher, respectively, than those of the XY335 variety (**Figures 7A, B** **)**. After HTS treatment, the anthers were deformed by bending, shrinking, and drying. The ALs of the ND372 and XY335 varieties decreased by 10.5% and 7.0% (**Figures 7C, D** **)**, and their AVs decreased by 27.4% and 37.9%, respectively, compared with those of the CK group. Further comparison of the phenotypic differences between the XY335 and ND372 varieties under the same HTS revealed that the ALs and AVs of the 335HTS treatment group were 18.3% and 32.5% lower, respectively, than those of the 372HTS group (**Figure 7I**). The cross-section of the anthers of the tassel of the two varieties (**Figures 7E–H**), resulted in high temperatures, causing the epidermal and middle layer cells of the anther wall to deform and arrange loosely, the tapetal cells to degenerate, the vascular bundle cells to arrange irregularly and become thinner, and the pollen grains in the flower chamber to scatter, and pollen to shrink, thereby clearly demonstrating the performance of the XY335 variety. Overall, HTS caused anther deformities and decreased ALs and AVs. The average of the two varieties decreased by 22.9% and 35.2% (3-year average), respectively, and the overall shape of the anthers of the heat-sensitive maize varieties changed.

#### 3.5.2 Pollen microstructure

**Figure 8:**
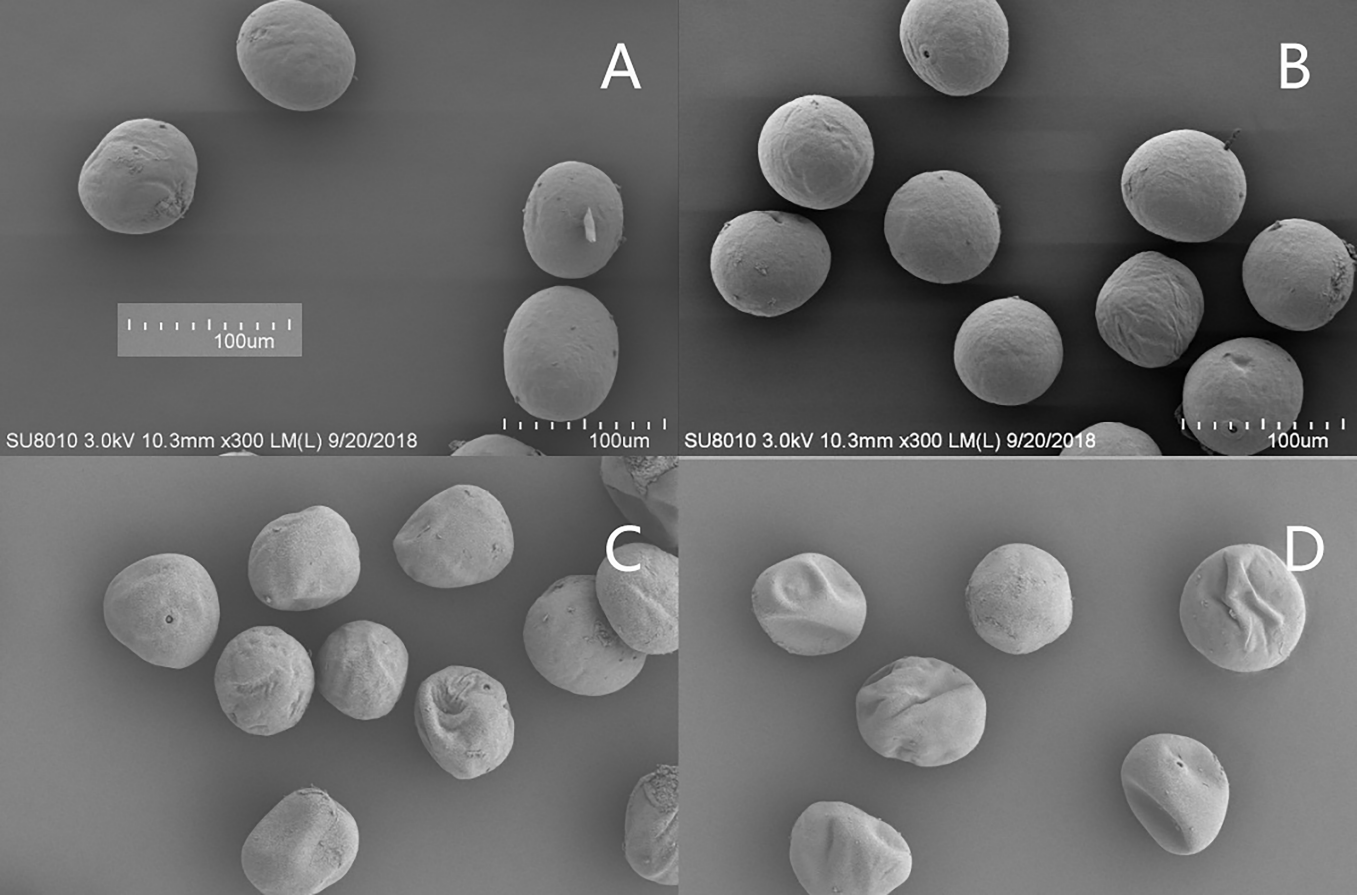
Surface ultrastructure of pollen at different temperatures (× 300). **(A, B)** are 372CK and 335CK, respectively; **(C, D)** are 372HTS and 335HTS, respectively.

Under natural conditions, the pollen of the two varieties was of regular morphology and the surface was smooth and almost free of wrinkles (**Figures 8A, B**). After the HTS treatment, the number of reticulated grains on the pollen surface of the XY335 variety increased and thickened, forming serious folds. However, under the same HTS conditions, the pollen surface reticulation of the ND372 variety either did not change significantly or was slightly thickened, resulting in the formation of a slight fold, and the degree of pollen aperture was lower than that of the XY335 variety (**Figures 8C, D** **)**. HTS changed the pollen diameter. Under natural conditions, the pollen diameter of the ND372 variety was larger than that of the XY335 variety; however, the difference between the varieties was not significant. After high-temperature treatment, there was a significant difference in pollen size between the two varieties, i.e., the pollen diameters of the ND372 and XY335 varieties decreased by 11.5% and 18.3%, respectively, compared with that of the control.

### 3.6 Effect of HTS on kernel number per ear and its correlation with tassels

**Figure 9:**
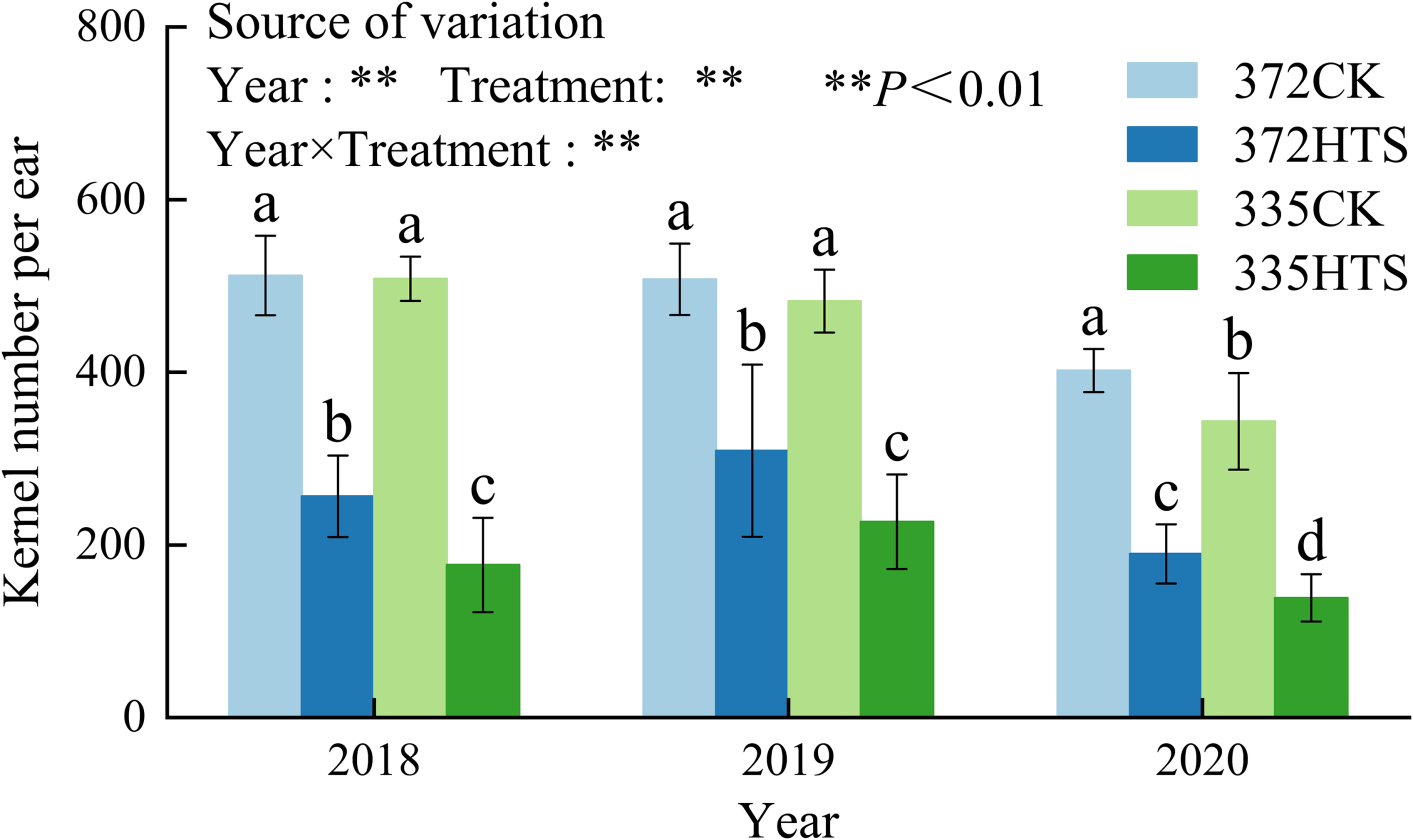
Kernel number per ear under different temperature. Different lowercase letters in the data indicate significant differences between different treatments in the same year (P<0.05).

**Figure 9** shows that HTS can reduce the kernel number per ear, and the range of decrease of different varieties is large. Under natural conditions, there was no significant difference in kernel number per ear between the two varieties in 2018 and 2019, and the kernel number per ear of the XY335 variety was significantly lower than that of the ND372 variety in 2020 (a decrease of 14.7%). HTS significantly reduced the kernel number per ear, and in the 372HTS and 335HTS varieties were decreased by 47.3% and 59.3%, respectively, compared with the corresponding CK. Further comparison of the grain number per ear of the two varieties under HTS revealed that the average grain number per ear of the 372HTS variety was 39.3% higher than that of the 335HTS variety (3-year average). The analyses of Y, T, and Y × T as a source of variation of treatment effect showed that all three had a highly significant effect on the kernel number per ear (*p *< 0.01; **Figure 9**).

**Figure 10:**
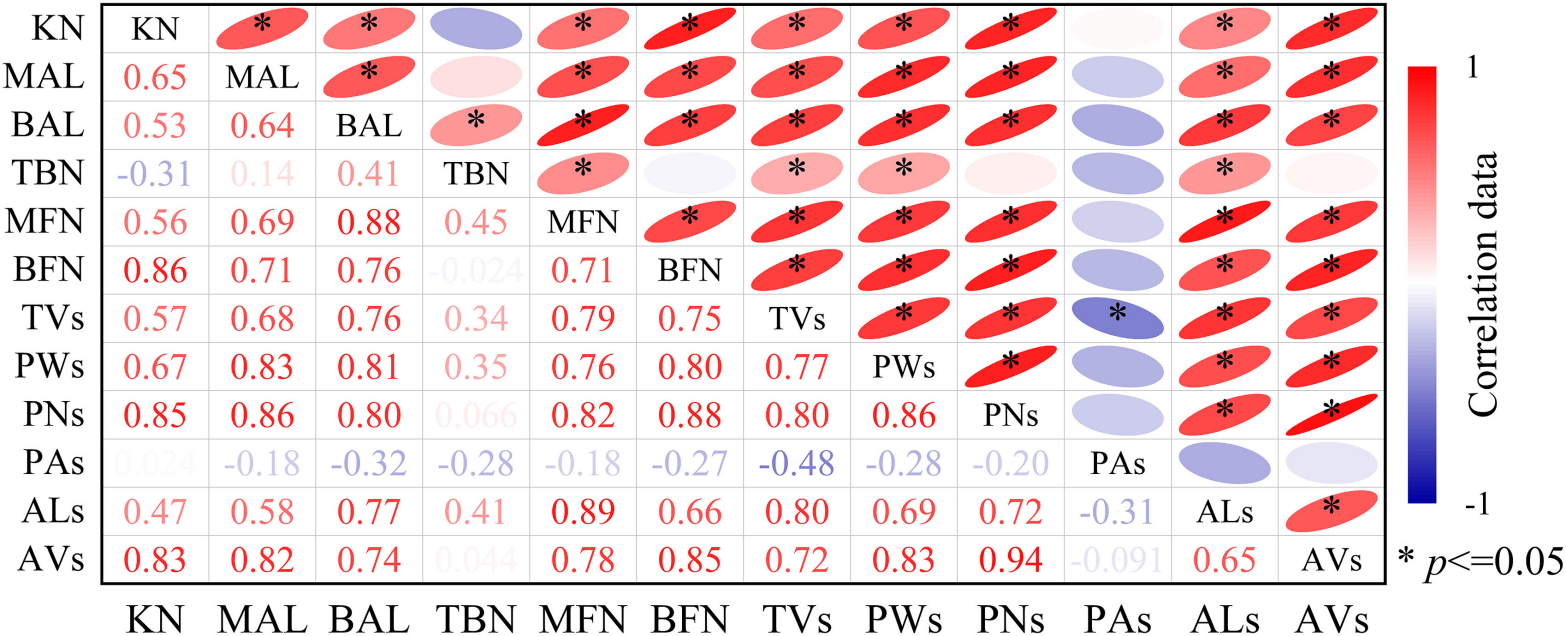
Correlation between the growth and development indexes of maize tassels and the kernel number per ear under different temperature. KN, kernel number per ear. In male spikes, MAL and BAL are the main axis and branch length, TBN is the number of branches, MFN and BFN are the number of main axis and branch florets, TVs is the volume, PWs and PNs are the total weight and number of pollen grains per plant, respectively, PAs is the average vitality of the pollen, and ALs and AVs are the length and volume of the anther, respectively. r is the Pearson correlation coefficient. *p < 0.05.

The kernel number per ear is related to varying degrees of tassel shape, the number and weight of pollen, pollen vitality, and other phenotypic traits. **Figure 10** shows that KN was significantly positively correlated with MAL, BAL, MFN, BFN, TVs, ALs, AVs, PWs, and vigor (PAs) (*p *< 0.05), and the correlation coefficient (*r*) was 0.72–0.94. Among them, the *r* of KN and PNs was the highest (0.94), followed by those of MAL, BFN, and PWs (0.80 < *r *< 0.90). The *r*-values for KN and ALs were the lowest (0.65). KN was weakly negatively correlated with TBN and PAs (*p *> 0.05). In general, the pollens number and weight, and MAL and BAL of the tassel had a significant effects on the KN per ear (*r *= 0.86), especially the pollens number (*r *= 0.94).

## 4 Discussion

### 4.1 Effect of HTS on the morphology and physiological characteristics of tassels

HTS thresholds exist at different stages of crop growth. If the threshold is exceeded, a series of morphological and physiological processes are affected, and eventually the yield is reduced (Cairns et al., 2012; Prasad et al., 2017). The tassel is one of the most important organs in maize plants. Tassel differentiation and development have a profound impact on yield, as they directly determine the quantity and quality of pollen. Tassels begin to develop after the joining stage. At this time, the impact of HTS on tassels is serious, and damage, once it occurs, cannot be reversed (Hatfield and Prueger, 2015). Because the tassel is sensitive to high temperatures, some studies have suggested that the outer membranes of the anther are very thin, and therefore that the high temperatures of the external environment can be easily transmitted to the anther, leading to anther deformity and damage to physiological function (Sinsawat et al., 2004; Hussain et al., 2006). In the NCP, the temperature during the reproductive development in maize is often higher than the optimal temperature for the maximum differentiation of spikes (25–32°C). This further increases the risk to tassels under temperature stress (Shao et al., 2021).

This study found that HTS increased the maximum TBN to some extent (by 9.8% and 30.0% compared with the CK treatment for the 372HTS and 335HTS varieties, respectively) and decreased the volume of tassels, number of main axes and branch florets, and lengths of the main axis and branch. However, HTS also accelerated tassel decay. Therefore, we believe that a high temperature before the heading stage accelerated development and increased the number of branches, but decreased the total volume of the tassels. This is consistent with previous research results (Shao et al., 2021). In addition, the rapid development of tassels affected the differentiation of florets, resulting in a significant reduction in maize florets under the HTS treatment.

### 4.2 Effects of HTS on anther, pollen morphology, and pollen vitality

Most studies have shown that heat stress reduces the global yields of major crops to a greater extent than other environmental stresses (Deryng et al., 2014; Zhao et al., 2017). This effect is a result of frequent heat stress and coincides with sexual reproduction (Hedhly et al., 2009). Maize is a monoecious species, the plants of which bear a tassel at the top and a pistillate flower in the middle (Begcy and Dresselhaus, 2017). The synchronous flowering of the male and female reproductive organs and sufficient active pollen are critical determinants of successful pollination (Herrero, 2003). Research has shown that, in maize, male reproductive organs are more sensitive than female reproductive organs to HTS (Rattalino Edreira et al., 2011; Lizaso et al., 2018) (Rattalino Edreira et al., 2011). If pollen is exposed to a temperature above 32.5°C for a long period, its germination rate will significantly decline with the extension of exposure time, and may even approach zero (Hussain et al., 2006; Akbar et al., 2017). Other studies have also revealed that pollen number, pollen viability, kernel number, and grain yield are significantly reduced by increases in temperature from 36/26°C to 40/30°C approximately 2 weeks before and after silking (Begcy et al., 2019; Wang et al., 2019). However, current research results on the relationship between pollen morphology and vitality are inconsistent. Some studies have suggested that HTS causes pollen surface deformities that further affect pollen vitality. However, other studies have indicated that there is no relationship between pollen surface deformities and pollen vitality (Tsou et al., 2015).

This study showed that HTS reduced PWs and PNs in maize, but there was no significant difference between the two varieties (the PWs of the ND372 and XY335 varieties decreased by 41.2% and 40.2%, respectively, and PNs decreased by 32.3% and 35.9%, respectively, 3-year average). HTS also reduced pollen vitality (the two varieties decreased by an average of 16%), and the decrease in the heat-sensitive varieties was greater. These results support the findings of previous studies (Hussain et al., 2006; Begcy et al., 2019; Wang et al., 2019). HTS also leads to surface deformation and a reduction in pollen diameter. High temperatures caused further deformities, in addition to decreases in the length and volume. The average of the two varieties decreased by 22.9% and 35.2%, respectively (3-year average), and the overall shape of the anthers of the high temperature-sensitive varieties increased. Notably, under HTS, the difference in pollen quantity between the maize varieties with different heat tolerances was not significant. The main differences were in anther and pollen quality, especially in terms of pollen phenotypic structure and vitality. This is consistent with results reported for other crops (Djanaguiraman et al., 2017). In addition, this study confirmed the findings of a previous study showing that the tassels of heat-sensitive varieties are more sensitive to high temperature (Wang et al., 2021).

### 4.3 Effect of HTS on kernel number per ear

The kernel number and weight are the determining factors of yield during the critical, approximately 30-day, period of silk formation (Cerrudo et al., 2020). However, the number of grains is not only determined after silking but also has a significant impact on the process of spike differentiation and pollination before silking. Therefore, some studies have indicated that temperatures during late vegetative growth and flowering greatly determine seed set (Wang et al., 2019). Many studies have shown that HTS at the flowering stage can significantly affect the number of kernels per ear, leading to a significant decline in the rate of seed setting (Alam et al., 2017; Prasad et al., 2017; Lizaso et al., 2018; Dong et al., 2021) and further affecting yield. This occurs after the V9 stage, when most of the male panicles are formed while the grains are still developing. HTS causes serious stress to both male and female reproductive organs, posing the hidden danger of a subsequent decline in kernel number per ear (Loussaert et al., 2017). Wang et al. (2019) reported that the kernel number per ear decreased by 77.6% in the group exposed to HTS compared with the conventional treatment group (temperatures were 40°C during the day and 30°C at night, and 32°C during the day and 22°C at night, respectively), 7 days before and after flowering. After HTS of the two heat-sensitive varieties, the bald tip length of the heat-sensitive variety (XY335) was significantly greater than that of the heat-resistant variety (ZD958), and the kernel number and yield per ear were significantly decreased (Shao et al., 2021).

This study showed that HTS significantly reduced the kernel number per ear, and in the ND372 and XY335 varieties it was decreased by 47.3% and 59.3%, respectively, compared with the CK of their respective varieties. Compared with the grain number per ear of the two varieties under HTS, the average grain number per ear of the 372HTS variety was 39.3% larger than that of the 335HTS variety (3-year average). The number and weight of pollen grains and the MAL and BAL of the tassel had significant effects on KN, particularly the number of pollen grains (*r *= 0.94). The effects of HTS on the shape of the tassel and the amount of pollen dispersed did not differ among the different varieties; however, HTS had significant effects on anther morphology, pollen vitality, and the phenotype of heat-sensitive varieties. In modern maize breeding, tassel reduction is a mainstream trend requiring the use of light energy and leading to reduced nutrient consumption in crop fields (Lu et al., 2015; Xu et al., 2017). However, a reduction of pollens number in the tassel leads to plants being incapable of coping with frequent HTS events in the future (Westgate et al., 2003).

## 5 Conclusion

HTS reduced the PNs, PWs, MAl and BAL of the tassel, which was the main reason for the reduced kernel number per ear. The effects of HTS on the shape of the tassel and the amount of loose pollen in maize did not differ among the different heat-sensitive maize varieties; however, the effects of high temperature on anther morphology, pollen vitality, and the phenotype of heat-sensitive maize varieties were significant.

## Data availability statement

The original contributions presented in the study are included in the article/supplementary material. Further inquiries can be directed to the corresponding author.

## Author contributions

WZ and LG conceived the project and set the scientific objectives. BY, SZ, and PL contributed to the preparation of field experiments and data acquisition. PL, BY, WD, JR, YW, and LG wrote the manuscript. All authors contributed to the article and approved the submitted version.
